# Bidirectional association between infectious gastroenteritis and inflammatory bowel disease: a population-based study

**DOI:** 10.1186/s40001-023-01324-y

**Published:** 2023-09-11

**Authors:** Kuan-Chieh Tu, Ru-Yi Yu, Yu-Hsuan Lin, Chih-Chiang Chien, Chin-Li Lu

**Affiliations:** 1 Department of Cardiology, Chi-Mei Medical Center, Tainan, Taiwan; 2 Graduate Institute of Food Safety, College of Agriculture and Natural Resources, South District, National Chung Hsing University, 145 Xingda Road, Taichung, 402 Taiwan; 3 Department of Nephrology, Chi-Mei Medical Center, Tainan, Taiwan; 4 Department of Food Science and Biotechnology, College of Agriculture and Natural Resources, National Chung Hsing University, Taichung, Taiwan; 5 Department of Post-Baccalaureate Medicine, College of Medicine, National Chung Hsing University, Taichung, Taiwan

**Keywords:** Inflammatory bowel disease, Ulcerative colitis, Crohn’s disease, Infectious gastroenteritis, Bidirectional association, Epidemiology

## Abstract

**Background:**

Intertwined association between infectious gastroenteritis (IGE) and inflammatory bowel disease (IBD) has not been investigated clearly. We aimed to examine the bidirectional association between IGE and IBD.

**Methods:**

A bidirectional study using the Taiwan National Health Insurance Research Database was designed. Through a case-control design, we identified 2899 new IBD cases during 2006–2017 and matched to 28,990 non-IBD controls. We used conditional logistic regression model to estimate odds ratios (OR) of IBD for previous IGE in different exposure time-windows within 5-years before IBD diagnosis and Poisson regression model to estimate incidence rate ratio (IRR) of subsequent IGE for IBD group to non-IBD group.

**Results:**

The mean age at the initial IBD diagnosis was 41 years. More IBD patients (21.49%) than controls (12.60%) had been exposed to IGE during > 6 months to 5 years before IBD diagnosis, the OR of IBD for IGE was 1.89 [95% confidence interval: 1.69–2.11]. Excess OR decreased as IGE exposure time before the index date increased. More IGE episodes were associated with additional increase in IBD risk (OR: 1.64, 2.19, 2.57, 3.50, and 4.57 in patients with 1, 2, 3, 4, and ≥ 5 IGE episodes, respectively). The IRR of having IGE for IBD group to non-IBD group was 2.42 before IBD diagnosis and increased to 5.74 after IBD diagnosis.

**Conclusions:**

These findings suggested an IGE-IBD bidirectional association. More attention is needed for physicians to develop preventive strategies and be aware of the higher risk of subsequent IGE in IBD patients.

**Supplementary Information:**

The online version contains supplementary material available at 10.1186/s40001-023-01324-y.

## Introduction

Inflammatory bowel disease (IBD), including ulcerative colitis (UC), Crohn’s disease (CD), and unclassified IBD, results from genetic susceptibility and environmental factors that cause an imbalance in immune response and gut microbiota [1]. IBD has been more prevalent in Western countries. However, IBD incidence has been rising in newly industrialized countries in the past decades, such as in Africa, South America, and Asia including Taiwan (annual percentage change: 4.8% and 4.0% for UC and CD, respectively) [2]. Its increasing morbidity also worsens social economic burden [3–5].

Infectious gastroenteritis (IGE), which causes dysbiosis, plays an important role in the pathogenesis of IBD [6]. An association of increased IBD risk with enteric infections has been reported in high IBD prevalence populations [7–13], particularly with bacterial gastroenteritis [8–11, 13]. An increasing number of previous gastroenteritis episodes may further elevate IBD risk, and the relevant time interval between an episode of gastroenteritis and IBD development may be greater than 10 years [15].

Theoretically, the use of glucocorticoids, immunosuppressants, and biological agents in diagnosed IBD patients may increase the risk of IGE episode [16, 17]. The prevalence of intestinal superinfections by enteropathogens among patients with active IBD ranged from 6% to 26.8% [18, 19]. *Clostridium difficile*, *Campylobacter* species, *E.coli*, and *Salmonella* species have been commonly reported in the flare-up IBD in previous studies [18–26]. However, disparities in methodology, including limited sample sizes, the lack of a comparison group, and varied proportions of CD and UC in the population, have limited a valid conclusion to elucidate the relationship between intestinal infections and diagnosed IBD. In addition, population-based studies that compare the IGE incidences before and after IBD diagnosis are warranted. Knowledge of the bidirectional relationship between IGE and IBD might help us understand the entire disease course and elucidate the intertwined causal-relationship between IGE and IBD

To our best knowledge, no previous study has retrospectively investigated the role of IGE in IBD development and, at the same time, prospectively examined the subsequent risk of IGE after IBD diagnosis. Therefore, we used a population-based claims dataset to determine whether patients with incident IBD were more likely to experience antecedent IGE episodes and have an even greater risk of developing subsequent IGE after IBD diagnosis

## Methods

### Data source

Data in this study were retrieved from the Health and Welfare Data Science Center (HWDC) in Taiwan. It included claims data of the Taiwan National Health Insurance Research Database (NHIRD) and the Database of Death Certificates in Taiwan from 2002 to 2017. The Taiwan National Health Insurance (NHI) is a centralized and compulsory health insurance system covering > 99% of Taiwan’s citizens. The NHIRD includes medical records of outpatient and inpatient visits, basic beneficiary data, and data in the Registry for Catastrophic Illness (RCI), they can be linked together by encrypted key variables. The personal information in the NHIRD is shielded, impeding specific individuals or institutions from being identified by data users. Once a patient is registered in the RCI, they are exempt from copayment for related medical services. Therefore, the medical records and examination data of cases registered in the RCI must be reviewed and approved by an expert committee in the NHI. This study was approved by the institutional review board (IRB) of the National Cheng Kung University Hospital (IRB number: A-EX-108-021).

### Data availability statement

The data underlying this article were provided by HWDC in Taiwan and cannot be shared publicly due to privacy of study participants. Data users must analyze these data on-site under permission and can only take out the statistical summarized tables or figures.

### Study design and study subjects

This study comprised two parts. The first part was a matched case–control study investigating the association between antecedent IGE and IBD development. The second part was a cohort study investigating the association between diagnosed IBD and the risk of subsequent IGE incidence.

In this case-control study, IBD cases were patients with newly diagnosed IBD registered in the RCI database from 2006 to 2017. The diagnosis codes of IBD included 555/K50 for CD and 556/K51 for UC in the International Classification of Diseases, the 9th/10th revision of Clinical Modification [ICD-9-CM/ICD-10-CM]. Physicians have to submit patients’ histopathological and endoscopic (or radiologic) examination data to the NHI, and IBD patients shall then be registered in RCI after approval by the NHI’s Expert Committee. We retrospectively reviewed outpatient and inpatient medical records for each IBD case and determined the initial IBD diagnosis date which was defined as the index date in our study.

Controls were selected by the incidence density sampling method and were matched to IBD cases by birth date, age, and sex. For each newly diagnosed IBD patient, we randomly selected 10 concurrent controls from NHI beneficiaries who were still alive and free of IBD on the index date of the matched case. The index date of each case was assigned as the index date of the matched controls. Using the dynamic sampling method, once a control was diagnosed with IBD later in life, this control was classified as an IBD case from the day of the initial diagnosis. To restrict confounding effects, we excluded individuals with a history of colorectal cancer before the index date in both cases and controls. The selection process of the study subjects was depicted in the flowchart in Additional file 1: Figure S1.

The same study subjects in the case-control study were also included in the cohort study. We classified IBD cases as the exposed group and non-IBD controls as the non-exposed group. We retrospectively and prospectively calculated the incidence of antecedent and subsequent IGE. The follow-up period started from the index date of IBD diagnosis and ended on December 31, 2017.

### IGE exposure

Medical visits for IGE were indicated by relevant diagnosis codes (ICD9-CM/ICD10-CM codes: 001-009/A00-A09) in the medical records. Inpatient and outpatient medical records for both cases and controls were traced back to identify IGE episodes that occurred within five years before their index date. Since multiple IGE episodes rarely occurred in a considerably short period, we collapsed multiple medical visits with an IGE diagnosis into one episode if the time interval between any two visits was ≤ 30 days. We investigated IGE episodes in five exposure time-windows to determine the critical time that might be most relevant to IBD risk. The five time-windows were ≤ 1 year, > 6 months to 1 year, > 1–3 years, > 3–5 years, and > 6 months to 5 years before the index date.

In this cohort study, we recorded subsequent IGE episodes after IBD (or index date) during the follow-up period and antecedent IGE episodes before IBD (or index date).

### Potential confounding factors

Other characteristics that potentially contributed to the risk of IBD and were likely to change the probability of IGE were considered potential confounding factors. In addition to demographic characteristics (age and sex), we collected socioeconomic data on monthly NHI-insured salaries, median family-income levels, and urbanization levels of residence districts to reflect individual and neighborhood socioeconomic status, clinical features including the Charlson Comorbidity Index (CCI) [27], and history of selected immune-related diseases, such as asthma, rheumatoid arthritis, autoimmune thyroiditis, vasculitis, and ankylosing spondylitis. The diagnostic codes were listed in Additional file 1: Table S1.

### Statistical analysis

Categorical variables were described with counts and percentages and compared using the Chi-squared test between cases and controls as well as between the UC and CD groups. Continuous variables were described with mean and standard deviation (SD) and compared using Student’s t-test. In the case–control design, we constructed univariable and multivariable conditional logistic regression analyses to estimate the crude and adjusted odds ratios (ORs) of IGE for cases compared to their matched controls in each exposure time-window. The ORs in multivariable analyses were adjusted for potential confounding factors, including monthly insured salary, median family income level, urbanization level, CCI, and history of selected immune-related diseases. Since age at index date and sex were tightly matched and excellently balanced in the case and control groups, these two variables were not adjusted. Subgroup analyses were performed for patients with CD and UC. As CCI is a composite index of overall illness severity, we also adjusted for certain specific CCI-component diseases based on statistical and clinical significance. Because of the low prevalence, the selected immune-related diseases were managed as a composite variable, demonstrating the history of having at least one immune-related disease.

For the analysis of the association of IBD with the risk of subsequent IGE, we estimated the incidence rates (IR) and 95% confidence intervals (CI) of IGE before (pre-IBD) and after (post-IBD) IBD diagnosis in IBD and non-IBD groups, assuming that IR followed a Poisson distribution. We adopted a Poisson regression model with an offset of observed person-years to estimate the adjusted incidence rate ratio (IRR) between the IBD-group and non-IBD groups for pre-IBD and post-IBD IGE, respectively. The Wald test was used to estimate the CIs of the regression coefficients and determine statistical significance. The IRRs in the multivariable analyses were also adjusted for potential confounding factors, as mentioned above in the logistic regression analyses. Data analyses in this study were performed using SAS/STAT software, Version 9.4 of the SAS system for Windows^©^ 2002–2012 (SAS Institute Inc., Cary, NC, USA).

## Results

### Demographics and clinical characteristics in IBD cases and controls

Table 1 showed the sociodemographic characteristics of IBD patients and controls. This study included 2899 IBD cases and 28,990 matched controls. The IBD cases were initially diagnosed at a mean age of 41 years. Sex and age at the index date of the cases and controls were well-balanced. IBD patients had a higher insured salary per month and lived in more affluent communities than the controls. Of the 2889 IBD patients, 923 (31.84%) had been exposed to IGE during the five years before the index date, while 3641 (12.56%) out of 28,990 controls had been exposed to IGE. As we excluded the IGE episodes occurring within 6 months before the index date, people with IGE exposure decreased to 520 (21.49%) and 3,048 (12.60%) among the cases and controls, respectively. UC (2,071 cases, 71.4%) was the dominant subtype of IBD in our study. Compared with CD patients, UC patients were more likely to be female, older, have a lower monthly insured salary, and were less likely to have a history of IGE during the 5 years before the index date in almost every study time-window.

**Table 1:** Sociodemographic characteristics and risk of infectious gastroenteritis in cases and controls

Characteristics	Controls N = 28,990	IBD Cases N = 2899	p-value	IBD Cases		p-value
				CD (N = 828)	UC (N = 2071)	
Sex						
Males	18,510 (63.85)	1851 (63.85)	1.000	563 (68.00)	1288 (62.19)	**0.003**
Females	10,480 (36.15)	1048 (36.15)		265 (32.00)	783 (37.81)	
Age (years), mean ± SD	41.10±15.83	41.10±15.83		36.83±16.60	42.80±15.18	
> 12–18	1890 (6.52)	189 (6.52)	1.000	98 (11.84)	91 (4.39)	**< 0.001**
19 to < 40	12,520 (43.19)	1252 (43.19)		422 (50.97)	830 (40.08)	
40 to < 60	10,800 (37.25)	1080 (37.25)		210 (25.36)	870 (42.01)	
≥ 60	3780 (13.04)	378 (13.04)		98 (11.84)	280 (13.52)	
Monthly insured salary, USD						
< 700 (median)	11,945 (43.38)	1052 (37.37)	**< 0.001**	275 (34.16)	777 (38.66)	**0.026**
≥ 700 (median)	15,591 (56.62)	1763 (62.63)		530 (65.84)	1233 (61.34)	
Median annual family-income in areas of residence (USD) ^a^						
< 17,000 (Q1)	6594 (24.82)	675 (25.26)	**0.021**	178 (23.30)	497 (26.05)	0.506
17,000 (Q1) to < 18,600 (Q2)	6629 (24.95)	631 (23.62)		188 (24.61)	443 (23.22)	
18,600 (Q2) to < 20,600 (Q3)	6717 (25.28)	635 (23.76)		183 (23.95)	452 (23.69)	
≥ 20,600 (Q3)	6628 (24.95)	731 (27.36)		215 (28.14)	516 (27.04)	
IGE ever occurred in 5 time-windows						
≤ 1 year	1026 (3.54)	609 (21.01)	**< 0.001**	199 (24.03)	410 (19.80)	**0.011**
> 6 months–1 year	543 (1.87)	161 (5.55)	**< 0.001**	59 (7.13)	102 (4.93)	**0.020**
> 1–3 years	1794 (6.19)	377 (13.00)	**< 0.001**	149 (18.00)	228 (11.01)	**< .001**
> 3–5 years	1638 (6.77)	227 (9.38)	**< 0.001**	80 (11.08)	147 (8.66)	0.061
> 6 months–5 years	3048 (12.60)	520 (21.49)	**< 0.001**	203 (28.12)	317 (18.67)	**< 0.001**

Digits present number (percentage), unless otherwise specified

Bold values represent p < .05

*IGE* infectious gastroenteritis. *IBD* inflammatory bowel disease

Table 2 compared the history of selected diseases between the groups. During the period > 6 months to 1 year before the index date, IBD cases were more likely than controls to have a history of asthma, rheumatoid arthritis, vasculitis, ankylosing spondylitis, and at least one of the selected immune-related diseases. Compared to CD patients, UC patients were less likely to have a history of rheumatoid arthritis, vasculitis, ankylosing spondylitis, and at least one of the selected immune-related diseases.

**Table 2:** Prevalence of immune-related diseases prior to one year before the index date

Immune-related diseases	Controls, n (%)	Cases, n (%)	p-value	Cases		p-value
				CD, n (%)	UC, n (%)	
At least one						
without	28,234 (97.39)	2777 (95.79)	**< 0.001**	783 (94.57)	1994 (96.28)	**0.038**
with	756 (2.61)	122 (4.21)		45 (5.43)	77 (3.72)	
Asthma						
without	28,628 (98.75)	2848 (98.24)	**0.020**	813 (98.19)	2035 (98.26)	0.892
with	362 (1.25)	51 (1.76)		15 (1.81)	36 (1.74)	
Rheumatoid arthritis						
without	28,913 (99.73)	2882 (99.41)	**0.002**	819 (98.91)	2063 (99.61)	**0.033**
with	77 (0.27)	17 (0.59)		9 (1.09)	8 (0.39)	
Psoriasis						
without	28,912 (99.73)	2886 (99.55)	0.084	824 (99.52)	2062 (99.57)	1.000
with	78 (0.27)	13 (0.45)		4 (0.48)	9 (0.43)	
Autoimmune thyroiditis						
without	28,968 (99.92)	2895 (99.86)	0.291	828 (100.00)	2067 (99.81)	0.583
with	22 (0.08)	4 (0.14)		0 (0.00)	4 (0.19)	
Vasculitis						
without	28,899 (99.69)	2879 (99.31)	**0.001**	818 (98.79)	2061 (99.52)	**0.033**
with	91 (0.31)	20 (0.69)		10 (1.21)	10 (0.48)	
Ankylosing spondylitis						
without	28,899 (99.69)	2878 (99.28)	**< 0.001**	817 (98.67)	2061 (99.52)	
with	91 (0.31)	21 (0.72)		11 (1.33)	10 (0.48)	**0.015**

Bold values represent p < .05

*CD* Crohn’s disease; *UC* ulcerative colitis

### IBD risk and IGE episodes

**Fig. 1:**
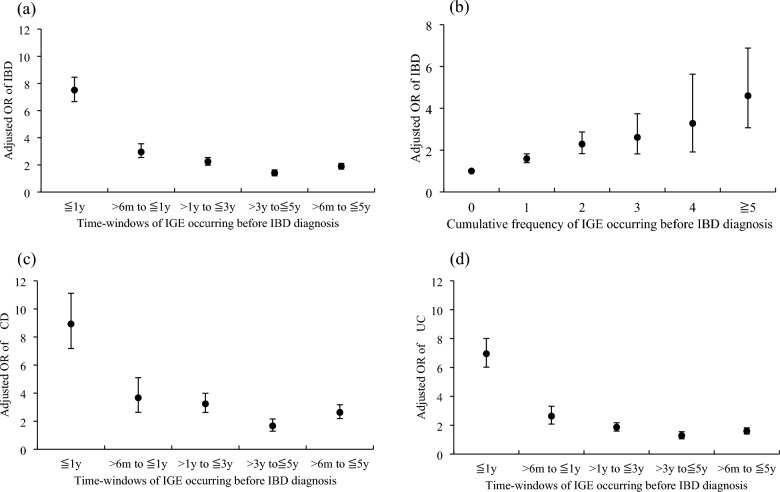
Adjusted ORs of risk of IBD associated with IGE. Adjusted ORs of risk of IBD associated with IGE occurred in 5 time-windows (**a**), cumulative frequency of IGE occurred during > 6m to ≤ 5y before IBD diagnosis (**b**), adjusted ORs of risks of CD associated with IGE (**c**), and adjusted ORs of risks of UC associated with IGE (**d**). Error bars represent 95% CI

Table 3 showed that IBD cases had increased odds of experiencing antecedent IGE within 1 year before the index date (adjusted OR [aOR]: 7.51). However, the aOR decreased to 2.95 as IGE episodes occurring shortly within 6 months before the index date were excluded. The aOR relating IBD risk to antecedent IGE from > 6 months to 5 years before the index date was 1.89 (95% CI 1.69–2.11). Excess odds of IGE in cases compared to controls were consistently observed in each exposure time-window. Compared to controls, IBD patients were more likely to have IGE during > 6 months to 1 year (aOR 2.95), > 1 to 3 years (aOR 2.24), and > 3 to 5 years (aOR 1.40) before the index date. The aOR strength decreased as the IGE exposure time increased before the index date (Fig. 1a). Similar trends were observed for both CD (Fig. 1c) and UC (Fig. 1d). In each study period, IGE was more prominently associated with CD than with UC.

**Table 3:** Crude and adjusted odds ratio for the risk of IBD associated with IGE occurred in the five time-windows

Time-windows prior to the index date	All IBD cases vs. controls			CD cases vs. controls		UC cases vs. controls	
	Crude OR (95% CI)	aOR (95% CI)^a^	p-value	aOR (95% CI)^a^	p-value	aOR (95% CI)^a^	p-value
≤ 1 year							
Without IGE	Ref.	Ref.		Ref.		Ref.	
With IGE	7.49 (6.70–8.38)	7.51 (6.66–8.46)	**< 0.001**	8.93 (7.18–11.11)	**< 0.001**	6.95 (6.02–8.01)	**< 0.001**
> 6 months–1 year							
Without IGE	Ref.	Ref.		Ref.		Ref.	
With IGE	3.07 (2.57–3.68)	2.95 (2.54–3.56)	**< 0.001**	3.67 (2.64–5.10)	**< 0.001**	2.63 (2.08–3.31)	**< 0.001**
> 1–3 years							
Without IGE	Ref.	Ref.		Ref.		Ref.	
With IGE	2.27 (2.02–2.55)	2.24 (1.98–2.54)	**< 0.001**	3.24 (2.63–3.99)	**< 0.001**	1.86 (1.59–2.17)	**< 0.001**
> 3–5 years							
Without IGE	Ref.	Ref.		Ref.		Ref.	
With IGE	1.43 (1.23–1.65)	1.40 (1.20–1.63)	**< 0.001**	1.67 (1.29–2.16)	**< 0.001**	1.28 (1.06–1.55)	**0.011**
> 6 months–5years							
Without IGE	Ref.	Ref.		Ref.		Ref.	
With IGE	1.91 (1.72–2.12)	1.89 (1.69–2.11)	**< 0.001**	2.63 (2.19–3.17)	**< 0.001**	1.59 (1.39–1.82)	**< 0.001**

Bold values represent p < .05

*IBD* inflammatory bowel disease; *CD* Crohn’s disease; *UC* ulcerative colitis; *IGE* infectious gastroenteritis; *OR*, odds ratio; *aOR* adjusted OR; *Ref.* reference group

^a^adjusted for monthly insured salary, median annual family-income level, urbanization level, Charlson’s comorbidity index, any history of selected immune-realted diseases, history of chronic pulmonary disease, rheumatic disease, peptic ulcer disease, mild liver disease, diabetes without chronic complications, and diabetes with chronic complications

We further examined the relationship between the cumulative frequency of IGE and IBD risk during the period > 6 months to 5 years before the index date. The ORs for IBD increased linearly with the frequency of antecedent IGE. Compared with people without a history of IGE, those who had experienced 1, 2, 3, 4, and 5 IGE episodes had increasing ORs of 1.64, 2.19, 2.57, 3.50, and 4.57, respectively. (Fig. 1b; p-for-trend < 0.0001).

### Bidirectional association between IGE and IBD

We further evaluated the bidirectional relationship between IGE and IBD. The incidence rates of IGE before and after the IBD index date were compared between IBD patients and controls (non-IBD group). Table 4 showed that IBD patients were more likely to experience pre-IBD IGE (138.8 vs. 55.7 per 1000 person-years) and post-IBD IGE (312.2 vs. 54.4 per 1000 person-years). IGE incidence did not significantly change before and after the index date in the non-IBD group but significantly increased 2.25 times in the IBD group. Compared with the non-IBD group, the adjusted IRR was 2.42 (95 % CI 2.34–2.51) before index date in IBD group. However, it increased to 5.74 (95 % CI 5.54–5.95) after the index date.

**Table 4:** Incidence rate of IGE episodes before and after IBD diagnosis in IBD cases and controls

	Controls			IBD cases		
	Before	After	p-value	Before	After	p-value
Mean number of IGE episodes	0.56 ± 2.48	0.32 ± 1.64	< 0.001	1.39 ± 3.40	1.83 ± 6.45	< 0.001
Total number of IGE episodes	16,152	9271		4022	5295	
Observed person-years	289,742.9	170,520.6		28,974.3	16,957.9	
Incidence rate, per 1000 person-years	55.7	54.4		138.8	312.2	
Crude IRR (95% CI)	Ref.	0.98 (0.95, 1.00)	0.055	Ref.	2.25 (2.16-2.34)	< 0.001
Adjusted IRR (95% CI)^a^	Ref.	Ref.		2.42 (2.34–2.51)^b^	5.74 (5.54–5.95)^c^	

*IGE* infective gastroenteritis. *IRR* incidence rate ratio. *Ref.* reference group

^a^Adjusted for monthly insured salary, median annual family-income level, urbanization level, Charlson’s comorbidity index, any history of selected immune-related disease

^b^compared to incidence rate of controls before the index date (p< 0.001)

^c^compared to incidence rate of controls after the index date (p< 0.001)

## Discussion

We used the national health claims database to identify IBD cases in Taiwan, most of whom had UC (71.4%). To our best knowledge, this is the first longitudinal study to suggest a bidirectional association between IGE and IBD. IBD patients had higher odds of experiencing IGE within five years before IBD diagnosis than controls, the OR increased with an increased cumulative frequency of IGE episodes. However, IBD cases had an even higher IRR of subsequent IGE after IBD diagnosis.

We found that the excess incidence and mean number of IGE episodes in IBD cases were magnified after IBD diagnosis. Compared with the controls, the IRR increased from 2.42 before IBD diagnosis to 5.74 after IBD diagnosis. In the aftermath of IBD, various factors such as gut dysbiosis, compromised repairment of the gut epithelial barrier, dysregulated immune responses, and persistent intestinal inflammation may collectively contribute to the rising incidence of IGE [28]. Retrospective cohort studies found that patients with IBD have a higher likelihood of experiencing various common infections, including gastrointestinal infections [28–30]. Dysbiosis of intestinal microbiota which had been described in the patients with IBD may activate innate immune system and is associated with mucosa damages [31]. Impaired mucosal barrier and increased paracellular permeability may enable penetration by bacteria and increase the risks of intestinal infections [32, 33]. In addition, the use of steroid and multiple immunosuppressant agents on the vulnerable mucosa of the chronic inflamed bowel may increase the risk of IGE after IBD diagnosis [16, 17]. Several studies have investigated the prognostic role of specific pathogens identified in IBD patients, particularly *Clostridium difficile*, *Cytomegalovirus*, *Campylobacter spp.*, and other pathogenic bacteria [18, 19, 21, 24, 26]. However, most of these studies were cross-sectional and only included inpatients during IBD flare-up, or lacked a healthy control group, limiting them from providing a comprehensive picture of the excessive IGE incidence before and after IBD diagnosis.

Our study revealed an association between previous IGE episodes and IBD development. A history of IGE occurring within 1 year before the index date was significantly associated with an increased IBD risk. This association diminished after excluding IGE within 6 months before IBD diagnosis, at which point the diagnosed IGE might be the initial presentation of IBD, according to the estimation from CHAD trial [12]. The association (aOR 1.89) between IGE and IBD diminished over time but remained statistically significant through the five-years before IBD diagnosis. A similar trend was observed in both the CD and UC cases. The study of Axelrad et al. had similar findings which demonstrated a previous gastroenteritis significantly associated with IBD at ≥10 years after the episodes (aOR 1.26, 95% CI 1.19–1.33) [15].

Our study also revealed that IBD risk increased with cumulative IGE episodes during > 6 months to 5 years before the index date. Compared to the control group, aOR increased from 1.64 to 4.57 in people experiencing 1 to ≥5 IGE episodes. These findings are similar to those from the study of Axelrad et al. [15], which reported an aOR of 1.61 to 4.64 corresponding to IGE frequency from 1 to > 4 before IBD diagnosis. Previous epidemiologic studies have linked specific intestinal pathogens to the increased risk of IBD development, the most reported included *Salmonella*, *Campylobacter*, and *Clostridium difficile*, while specific pathogens could only be identified in quite limited IGE episodes in IBD cases [7, 8, 10, 15], and potential detection bias resulted from more stool tests in IBD group may compromize the causal relationships between sepecific pathogens and IBD development [14].

Intestinal microbiota are important and necessary for humans, as they participate in the process of intestinal homeostasis, intestinal immune development, host metabolism, and protection of the host from pathogenic infection via colonization resistance [34–36]. The coexistence of intestinal microbiota is maintained by various immune mechanisms, including intestinal mucus secretion, immunoglobulin A, and antimicrobial peptides, which shape the gut microbiota and prevent direct contact with the epithelium [37]. IGE disrupt the equilibrium of the gut microbiota and elicit a robust immune response against invasive pathogens. As a consequence, this immune reaction can disturb the microbial balance, resulting in dysbiosis that has been associated with the initiation and progression of IBD [38]. Numerous animal models have indicated that colitis led to a compromised immune response (loss of tolerance to commensals and microbiota-specific T cell differentiation) and chronic inflammation of the intestine, corroborating the notion of dysregulated immunity as a key driver of IBD [39, 40]. The imbalance between commensal and pathogenic microorganisms may promote IBD development [34, 35]. In-vitro studies have suggested the role of enteric infections in promoting gastrointestinal microbial dysbiosis and subsequently causing characteristics of intestinal inflammation of IBD [41–44].

The present study had certain limitations. First, this study lacked sufficient culture data or specific pathogen diagnoses for most gastroenteritis events, which may limit our study to further associate IBD risk with specific pathogens and distinguish between IGE episodes and IBD-related symptoms. Second, there are also some limitations that may cause misclassification of IGE in our study. The IGE diagnosis was based on ICD codes in our study. Without information on the clinical presentations of each IGE episode, there may be misclassification of IGE in both the IBD group and the non-IBD group. In addition to that, the number of IGE cases may be underestimated because many people with IGE did not seek medical care. Moreover, many people who were eventually diagnosed with IBD initially presented with some digestive symptoms and were diagnosed as having IGE. Therefore, we compared the frequency of IGE between two groups in several time-windows as early as possible before IBD was diagnosed (i.e., IGE occurred in > 6 months to 1 year, > 1–3 years, > 3–5 years before the index date). This minimized the possibility of unequal frequency of seeking medical care for IGE between IBD cases and controls, it also largely excluded the IGE-like symptoms which were essentially early manifestations of IBD. As a result, the non-differential misclassification of IGE between the two groups could only lead to the association between IBD and IGE being underestimated and towards the null.

In conclusion, our study suggested a bidirectional association between IGE and IBD. The patients with incident IBD had experienced more IGE previously and were more likely to have subsequent IGE after IBD development. These findings highlight the potential impact of IGE and alert physicians to be aware of the increased risk of subsequent IGE in IBD patients, especially in newly industrialized countries where IBD incidence has been markedly increased in the past decades.

### Supplementary Information

**Additional file 1: Figure S1.** Flow chart for study subject selection. **Table S1.** ICD codes of immune-related diseases.

## Data Availability

The data that support the findings of this study are available from the Health and Welfare Data Science Center in Taiwan but restrictions apply to the availability of these data, which were used under license for the current study, and so are not publicly available.

## Abbreviations

IBD: Inflammatory bowel disease
UC: Ulcerative colitis
CD: Crohn’s disease
IGE: Infectious gastroenteritis
HWDC: Health and Welfare Data Science Center
NHIRD: Taiwan National Health Insurance Research Database
NHI: National Health Insurance
RCI: Registry for catastrophic illness
IRB: institutional review board
ICD-9-CM: International Classification Of Diseases, The 9th Revision Of Clinical Modification
CCI: Charlson comorbidity index
IR: Incidence rate
OR: Odds ratio
CI: Confidence interval
IRR: Incidence rate ratio
