# Supplementary material for: Bidirectional association between infectious gastroenteritis and inflammatory bowel disease: a population-based study
Source: Eur J Med Res. 2023 Sep 11;28:337. doi: 10.1186/s40001-023-01324-y (PMC10494362; doi:10.1186/s40001-023-01324-y)
Supplement: Supplementary file 1 — Additional file 1: Figure S1. Flow chart for study subject selection. Table S1. ICD codes of immune-related diseases. [file 40001_2023_1324_MOESM1_ESM.docx]

Additional Materials

**Additional file 1: Figure S1. Flow chart for study subject selection.**


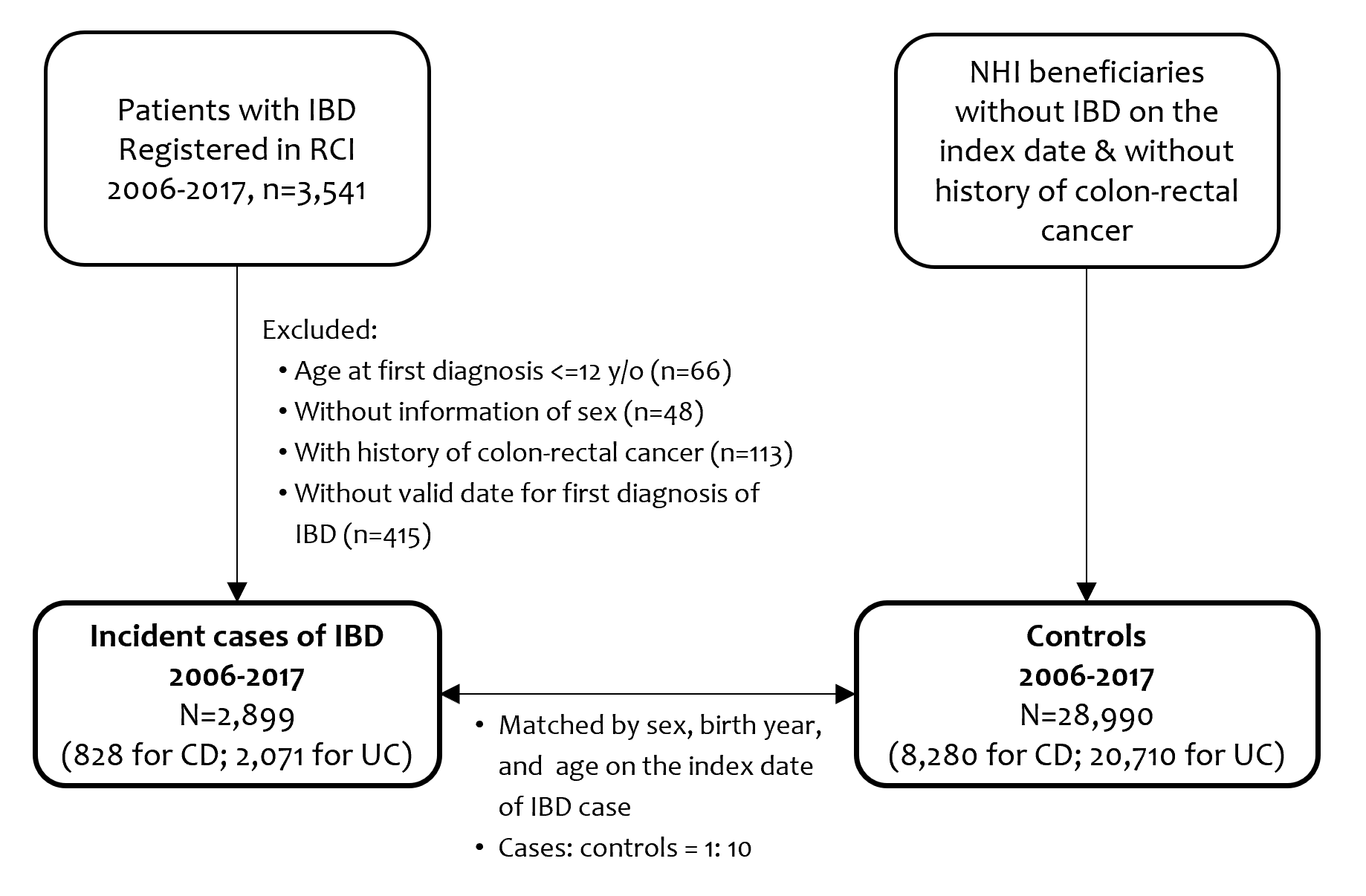


**Additional file 1: Table S1. ICD codes of immune-related diseases.**

|  | **ICD9-CM codes** | **ICD10-CM codes** |
| --- | --- | --- |
| Asthma | 493 | J45 |
| Rheumatoid arthritis | 714 | M05, M06, M08, M09 |
| Psoriasis | 696 | M07, L40, L41 |
| Multiple sclerosis | 340 | G35 |
| Autoimmune thyroiditis | 245 | E06.3 |
| Type 1 diabetes mellitus | 250.01, 250.03, 250.11, 250.13, 250.21, 250.23, 250.31, 250.33, 250.41, 250.43, 250.51, 250.53, 250.61, 250.63, 250.71, 250.73, 250.81, 250.83, 250.91, 250.93, 357.2, 366.41, 583.81 | E10 |
| Vasculitis | 446, 710 | M30, M31, M32, M33, M34, M35, M36 |
| Ankylosing spondylitis | 720 | M08.1, M45, M46.0, M46.1, M46.5, M46.8, M46.9, M48.8, M49.8 |

ICD-9(10)-CM, International Classification of Disease, Ninth (Tenth) Edition, Clinical Modification.
